# Case Report: Clinical benefit from multi-target tyrosine kinase inhibitor and PARP inhibitor in a patient with cancer of unknown primary with *BRCA1* large genomic rearrangement

**DOI:** 10.3389/fphar.2023.997760

**Published:** 2023-01-23

**Authors:** Ling Yu, Jietao Lin, Hanhan Li, Lingling Sun, Shubo Wang, Yaoxu Chen, Hanrui Chen, Lizhu Lin

**Affiliations:** ^1^ Department of Oncology, The First Affiliated Hospital of Guangzhou University of Chinese Medicine, Guangzhou, China; ^2^ The Medical Department, 3D Medicines Inc., Shanghai, China

**Keywords:** cancer of unknown primary, anlotinib, BRCA1, large genomic rearrangement, olaparib

## Abstract

**Background**: Cancer of unknown primary (CUP), which accounts for 3%–5% of new cancer cases every year, involves the presence of a type of histologically confirmed metastatic tumors whose primary site cannot be confirmed by conventional diagnostic methods. This difficulty in identifying the primary site means that CUP patients fail to receive precisely targeted therapy. Most patients are treated with empiric chemotherapy, with a median survival of 6 months and even poorer prognosis within an unfavorable subset of CUP.

**Case report:** An 80-year-old woman presented with masses in the abdomen. Following comprehensive imagological and immunohistochemical examinations, she was diagnosed with CUP. She emphatically declined chemotherapy; thus, anlotinib has been administered with patient consent since 02/07/2019, and stable disease (SD) was observed for 2 years. During subsequent treatment, a large genomic rearrangement in *BRCA1* was identified in the patient *via* NGS, and SD was observed for a further 6 months following olaparib treatment. The type of LGR identified in this patient was discovered to be *BRCA1* exon 17-18 inversion (inv), which has never been previously reported.

**Conclusion:** For CUP patients, a chemo-free regimen seems to be acceptable as a first-line treatment, and NGS-guided targeted treatment could improve patient outcomes.

## Introduction

Occult primary tumor, or cancer of unknown primary (CUP), which accounts for 3%–5% of new cancer cases every year, involves the presence of a type of histologically confirmed metastatic tumors whose primary site cannot be confirmed by conventional diagnostic methods (Pavlidis et al., 2003). Because of the difficulty in identifying the primary origin of these heterogeneous tumors, most patients are treated with empiric chemotherapy, with a median survival of 6 months and even poorer prognosis among patients in an unfavorable subset of CUP (Pavlidis et al., 2015; Yulian et al., 2022). A commonly utilized systemic chemotherapy regimen is carboplatin combined with paclitaxel (Laprovitera et al., 2021). However, newly launching targeted drugs offer increased opportunities for CUP patients harboring specific genomic alterations to receive molecularly guided therapy (Qaseem et al., 2019). In fact, 85% of CUP patients have been found to harbor one or more clinically relevant genomic alterations, which means that these patients are likely to receive personalized therapy (Ross et al., 2015). *BRCA1/2* is one of the most frequent genomic alterations in CUP (Ross et al., 2015). *BRCA1/2* serve as key genes in homologous recombination repair, suppress genome instability, and are also tumor suppressors (Smith and Pothuri, 2022). Homologous recombination deficiency (HRD) attributable to *BRCA1/2* mutation may lead to defective repairs to DNA damage; the mutations accumulating in this way may cause any form of cancer (Friebel et al., 2014; Vietri et al., 2021; Woodward and Meyer, 2021). Large genomic rearrangement (LGR) is a rare type of genomic alteration in *BRCA1/2* that is responsible for between 0% and 27% of all disease-causing *BRCA1/2* mutations identified (Sluiter and van Rensburg, 2011). To date, 120 LGRs in BRCA1 and 40 LGRs in *BRCA2* have been reported (Sluiter and van Rensburg, 2011). PARP inhibitors (PARPi), such as olaparib and niraparib, are a new set of options for cancer patients with *BRCA1/2* mutation that take effect through synthetic lethality to cancer cells with *BRCA1/2* mutation (Helleday, 2011). Several clinical studies have demonstrated that patients with ovarian cancer or prostate cancer carrying germline or somatic *BRCA1/2* mutation can achieve significant curative effects when treated with PARPi (Wu et al., 2021; Harter et al., 2022; O'Cearbhaill et al., 2022). Although the mechanism of PARPi is rather clear, no case report has been published on the use of PARPi in CUP patients harboring BRCA1/2 mutation. Herein, we present the case of an 81-year-old woman with CUP with BRCA1 exon 17-18 inversion, a previously unreported type of LGR, who has derived clinical benefit from treatment with anlotinib and PARPi.

## Case report

**FIGURE 1:**
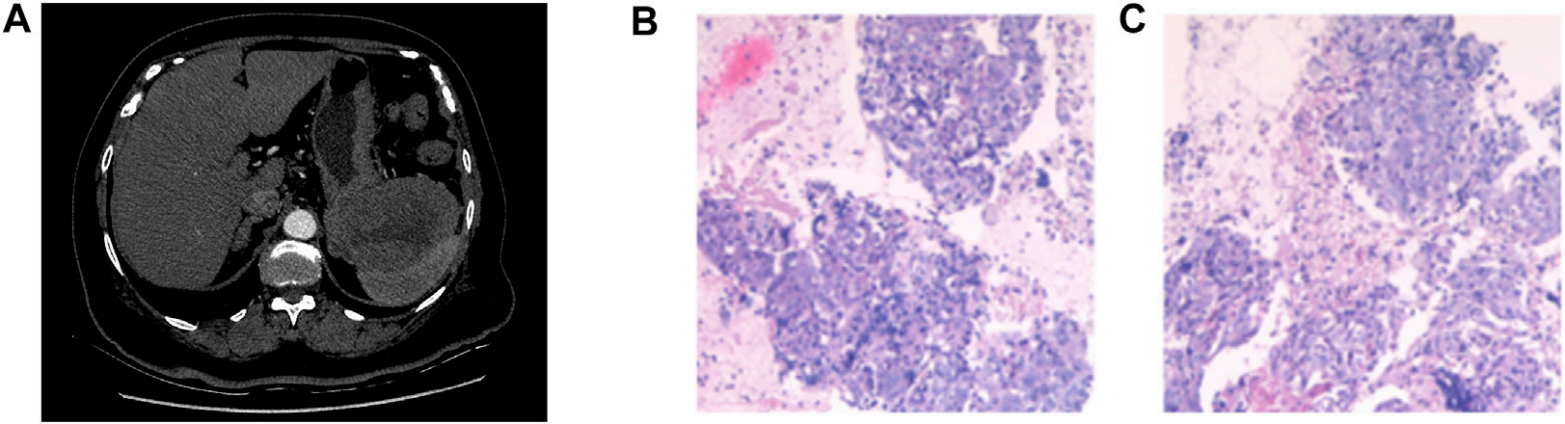
CT scan on 31 May 2019 revealed multiple masses in the gastrosplenic recess, the inferior capsule border of the spleen, and the right peritoneum **(A)**. Pathology results revealed poorly differentiated metastatic adenocarcinoma **(B, C)**.

An 80-year-old woman was admitted to the hospital with abdominal pain. She had a history of hypertension and diabetes for more than 10 years. During these 10 years, she took medication regularly, and her blood pressure and blood sugar levels were controlled within the normal range. She was also taking aspirin to prevent cerebral thrombosis. She had no family history of cancer. Her ECOG PS score was 2. The results of laboratory tests indicated normal levels of CEA, CA199, APF, and CA153; however, CA125 was elevated to 62.23 U/mL. A CT scan on 31 May 2019 revealed multiple masses in the gastrosplenic recess, the inferior capsule border of the spleen, and the right peritoneum (Figure 1A). In addition, localized thickening in the right peritoneum, soft tissue density shadow in several masses, and a small amount of fluid accumulation in the pelvic cavity were observed. The diagnosis on the basis of imaging results was considered to be peritoneal metastasis of malignant tumor. Biopsy of the masses in the gastrosplenic recess indicated poorly differentiated metastatic adenocarcinoma as the differential diagnosis (Figures 1B,C). Immunohistochemical examination was negative for CK20, PAX8, Vimentin, Syn, CgA, CR, HepPar-1, CDX2, S-100P, AFP and Desmin; CK7, CK8/18, and CD56 were positive; and P53, WT-1, and Ki67 (less than 10%) were positive in some cells. Although the results of imaging, immunohistochemistry, and serum markers suggested that the primary site of origin of the metastases may be an upper gastrointestinal tract carcinoma or ovary, further testing did not reveal the primary site. The patient was eventually diagnosed with metastatic cancer of unknown primary.

**FIGURE 2:**
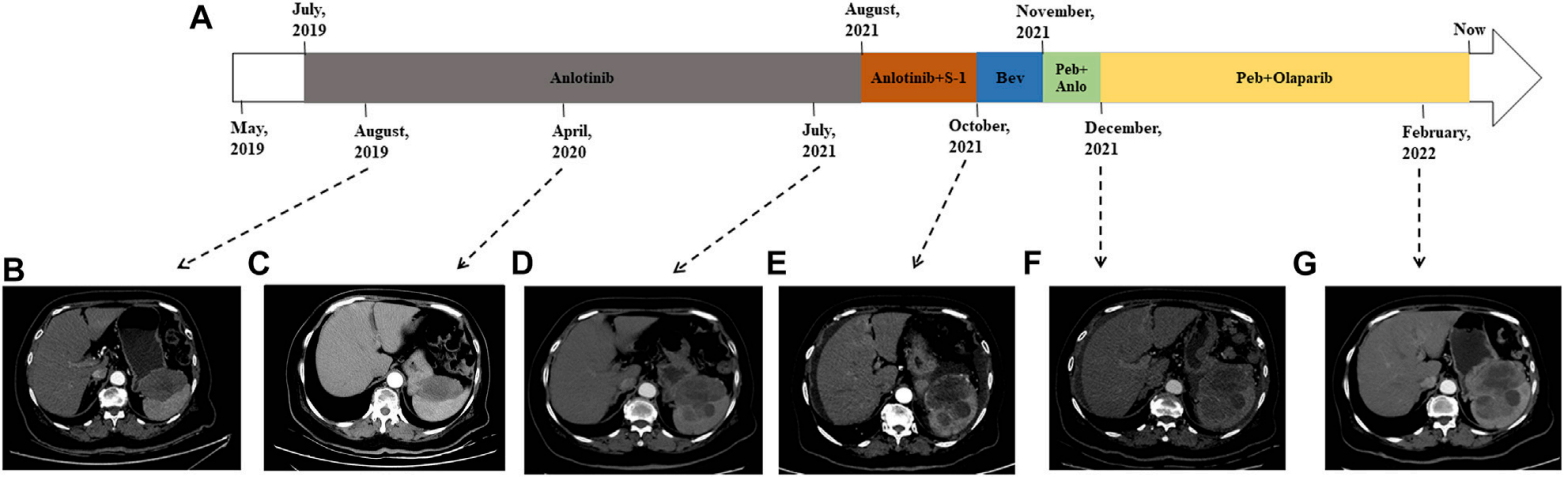
Treatment course of the patient **(A)**. CT scans in August 2019 **(B)** and April 2022 **(C)** indicated that the patient was experiencing SD. CT scan on 16 July 2021 revealed that the patient was experiencing PD **(D)**. CT scan on 1 October 2021 indicated that masses in the peritoneum and gastrosplenic recess were enlarged and had begun to further invade the spleen **(E)**. PD was observed according to CT scan on December 21 **(F)**. CT scan on 22 February 2022 indicated that the masses had slightly regressed and a decrease in ascites was observed **(G)**. Bev: bevacizumab; Peb: pebolizumab; Anlo: anlotinib.

**FIGURE 3:**
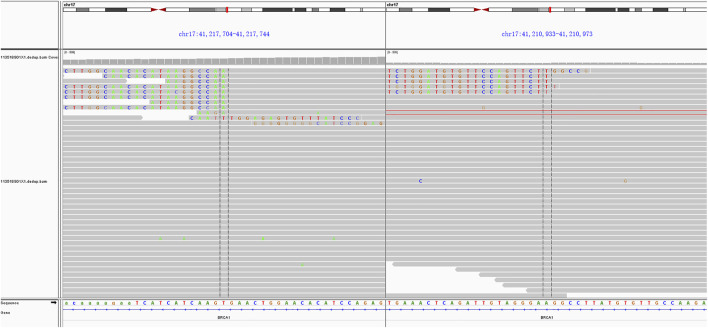
NGS indicated a somatic mutation: somatic *BRCA1* exon 17-18 inv.

**FIGURE 4:**
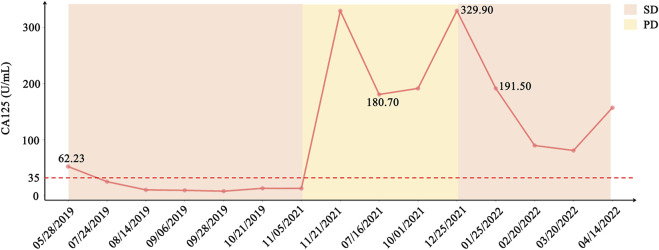
Variation in CA125 and status of the patient from initial treatment to current treatment. CA125 level decreased sharply after treatment with anlotinib in May 2019 and treatment with PARPi combined with pebolizumab in December 2021.

In consideration of her age and physical condition, we decided to offer the patient systemic therapy rather than debulking surgery (Figure 2A). In addition, the patient emphatically declined chemotherapy; thus, from 02 July 2019, anlotinib (12 mg, 6 cycles) was administered with the patient’s consent. During this treatment, the patient developed stomach discomfort, nausea and vomiting, and other adverse reactions, which were relieved after the dose was reduced to 8 mg. The patient experienced stable disease (SD) and CA125 returned to a normal level (Figures 2B,C). A CT scan on 16 July 2021 revealed that the masses in the gastrosplenic recess were enlarged compared to their size on the previous scan (Figure 2D). The patient was experiencing progressive disease (PD), and treatment with anlotinib and S-1 was commenced. After two cycles of this regimen, a CT scan on 1 October 2021 showed that the masses in the peritoneum and in the gastrosplenic recess had further enlarged and had begun to further invade the spleen (Figure 2E). Moderate ascites was observed. Bevacizumab was administered *via* intraperitoneal injection on October 15, and at the same time, a fresh ascites sample was obtained in order to conduct next-generation sequencing (NGS) analysis; this was carried out based on a pan-cancer 733-gene panel in 3D Medicine (Shanghai, China). Several mutations were identified in the sequencing results. The results suggested that this patient harbored somatic *BRCA1* exon 17-18 inv (Figure 3), and the tumor mutation burden (TMB) status of the samples was assessed as high (TMB-H). From November 6, treatment with anlotinib plus pebolizumab (2 cycles) was administered; however, PD was observed according to the results of a CT scan on December 21 (Figure 2F). During this treatment, the patient’s CA125 level increased from 180.70 U/mL to 329.90 U/mL. Eventually, on December 28, we altered the regimen to olaparib combined with pebolizumab. After 3 cycles of this regimen, a CT scan on 22 February 2022 indicated that the masses in the peritoneum and gastrosplenic recess had slightly regressed, and a decrease in ascites was observed (Figure 2G). Additionally, CA125 was reduced to 191.5 U/mL (Figure 4). At the time of writing, the patient’s survival with SD has already exceeded 6 months, and close follow-up is continuing.

## Discussion

Thanks to improvements in diagnostics, the percentage of patients diagnosed with CUP is decreasing year on year. However, the primary tumor site cannot be identified in all patients, and indeed the primary tumor is not identified even during autopsy in 70% of CUP patients (Pavlidis et al., 2003). Precisely tailored therapy cannot be offered to CUP patients in whom the primary tumor site cannot be identified, and the NCCN guidelines recommend chemotherapy as the standard of systemic therapy for these patients. A phase II clinical trial conducted by G Huebner *et al.* has revealed that the median overall survival (OS) is 11.0 months and the response rate is 23.8% among CUP patients treated with paclitaxel and carboplatin (Huebner et al., 2009). Unfortunately, the patient in the present case falls into the subset of CUP patients with unfavorable prognostic factors; patients in this category have poorer prognosis, with median OS of 3–10 months (Losa et al., 2018). For these patients, chemotherapy is recommended, although the likelihood that they will benefit from this is questionable. Thus, the exploration of new treatment options for CUP patients is an urgent priority.

Small-molecule multi-target tyrosine kinase inhibitors (SM-TKIs), such as anlotinib and sorafenib, are currently regarded as potential treatments for multiple advanced malignances. The mechanism includes targeting of multiple angiogenetic factor receptors in order to suppress angiogenesis and inhibition of some of the functions of tumor cells (Gao et al., 2020). The results of a phase III randomized controlled trial reported by Yihebali Chi et al. (2021) indicated that patients with refractory metastatic colorectal cancer who were treated with anlotinib exhibited improved progression-free survival (PFS) and overall response rate (ORR) over those treated with placebo. Patients with other tumors also experience significant survival benefits following treatment with SM-TKIs. Vasilis Karavasilis et al. (2005) have reported that angiogenesis is very active in CUP, and VEGF expression is high in 83% of patients with CUP). C Massard et al. (2007) have also discovered that EGFR and c-Kit expression can be observed in 66% and 10% of patients with CUP, respectively. These findings suggest that SM-TKIs are a promising regimen for CUP. A case report by Jingxian Chen et al. (2020) describes a case of CUP that was treated with sorafenib, in which SD was observed for 3 months . In the current case, SD has been observed in a patient treated with anlotinib for over 2 years.

After multiple regimens had failed, we carried out NGS analysis using ascites. One retrospective analysis has revealed that CUP patients may not derive clinical benefit from molecularly guided treatment approaches compared with standard treatment options (Fusco et al., 2022). However, this represents an alternative option for patients who are intolerant to chemotherapy or who decline chemotherapy, as did the patient in this case. The NGS results revealed somatic *BRCA1* exon 17-18 inversion, which is a rare type of LGR. The term LGR usually refers to the duplication or deletion of hundreds to millions of fragments, which may involve one or more exons, and most of these are deletions of gene fragments. In the case of exon 17-18 inversion, two breaks occur in the *BRCA1* gene, and the resulting fragments are reversed 180° and then re-spliced. Inversion differs from deletion or duplication, and is a form of balanced alteration. Because this genomic alteration involves two exons in the coding region, it is considered to disable BRCA1 protein. Clinical trials such as PAOLA-1 and PRIMA have demonstrated that patients with BRCA1/2 mutations can benefit from PARPi (Harter et al., 2022; O'Cearbhaill et al., 2022). In addition, Xiaomeng Jia *et al.* reported on a 63-year-old female CUP patient harboring *BRCA1* R71K mutation, in whom a partial response was observed lasting 15 months after treatment with olaparib as a third-line regimen (Jia et al., 2021). In the present case, the patient has experienced SD for 6 months after PARPi treatment. Although the patient was treated with olaparib combined with pembrolizumab based on the results of biomarker analysis (TMB-H), we believe that the patient’s clinical benefit may be primarily attributed to olaparib, because she did not benefit from a prior regimen of pembrolizumab and anlotinib. However, we cannot eliminate the possibility of an interaction between PARPi and immunotherapy. Jianfeng Shen et al. (2019) have revealed that PARPi may trigger the STING-dependent immune response, thereby enhancing the efficacy of immune checkpoint blockade. Moreover, the patient also harbored other genomic alterations, such as FAAP100; we cannot ignore the impact of these alterations on the efficacy of olaparib or anlotinib. Alteration of *FAAP100* in the Fanconi anemia (FA) pathway can also affect genome stability in the cells. It is unclear whether the presence of alterations to *FAAP100* and *BRCA1* synergistically enhances the efficacy of PARPi, and thus whether the efficacy of PARPi will not be much in evidence if there is only alteration to *BRCA1*; these are questions worthy of consideration. However, in this case, the mutation of FAAP100 (p.S688R) was classified as a variant of unknown clinical significance (VUS), and we believe that the clinical benefit of PARPi in this patient can be primarily attributed to the presence of *BRCA1* exon 17-18 inv. Given the nature of any case report, the conclusions that CUP patients may benefit from anlotinib treatment and that CUP patients with *BRCA1/2* mutations may benefit from PARPi should be further investigated in larger cohort studies.

## Conclusion

In conclusion, we have presented the case of a CUP patient who has achieved PFS for an impressive duration following treatment with anlotinib as a first-line regimen. During subsequent treatment, *BRCA1* LGR was identified in the patient *via* NGS, and SD was observed for a further 6 months following olaparib treatment. This case also reports a type of LGR that has never been previously reported.

## Data Availability

The original contributions presented in the study are included in the article/Supplementary Material, further inquiries can be directed to the corresponding authors.
